# Evaluation of the Effect of the 47 kDa Protein Isolated from Aged Garlic Extract on Dendritic Cells 

**Published:** 2012

**Authors:** Namdar Ahmadabad Hasan, Mohammad Hassan Zuhair, Elahe Safari, Mahmood Bozorgmehr, Seyed Mohammad Moazzeni

**Affiliations:** 1 *Department of Immunology, School of Medicine, Tarbiat Modares University, Tehran, Iran*

**Keywords:** Dendritic Cells, Garlic Extract, Maturation Markers

## Abstract

**Objective(s):**

Garlic (*Allium sativum*) is known as a potent spice and a medicine with broad therapeutic properties ranging from antibacterial to anticancer, and anticoagulant. One of the major purified garlic protein components is the 47 kDa protein. In this study, the effect of 47 kDa protein extracted from aged garlic (AGE) was evalua

**Materials and Methods:**

Forty seven kDa protein was purified from AGE by ammonium sulfate precipitation and gel filtration. SDS-PAGE was used to determine the molecular weight and purity of the isolated protein. DCs were purified from spleen of BALB/c mice by Nycodenz centrifugation and their adhesiveness to the plastic dish. The 47 kDa protein isolated from AGE was added to DCs medium during the overnight culture and the expression of DC surface markers was assessed via flowcytometry.

**Results:**

The 47 kDa protein-treated DCs lowered the expression of DC maturation markers including: CD40, CD86 and MHC-II in comparison with non-treated DCs; (median of 41% versus 47%, 84% versus 91% and 83% versus 90%, respectively) but we observed no statistical difference between the two groups.

**Conclusion:**

Upon treatment with DCs with 47 kDa protein, DCs down regulated the expression of costimulatory and MHC-II surface molecules, which is similar to tolerogenic DC phenotype. According to the results of the present study, we found that 47 kDa protein purified from AGE can be considered as a potential candidate to generate tolerogenic DCs *in vitro*.

## Introduction

Garlic (*Allium* *sativum*) is one of the oldest medicinal plants used by different cultures. In old days, it was used for treatment and prevention of some diseases (1). Garlic and its organosulfur compounds have been shown to reduce risk factors for cardiovascular diseases (2-4), and to suppress cancer cell growth *in vitro* (5) and *in vivo* (6,7). Furthermore, garlic has been shown to be a possible immune response modifier (4). According to our previous studies, it has been demonstrated that garlic enhances natural killer (NK) activity (8) and T-lymphocyte proliferation (9). Also, garlic extract and a garlic protein were shown to augment the oxidative burst in peritoneal macrophages of BALB/c mice (4). Ghazanfari *et al* showed that garlic extract induces a shift in cytokine pattern in *Leishmania major*-infected BALB/c mice (10) leading to T helper 1 (Th1) immune responses (IFN-γ, IL-2).

A unique garlic preparation called “aged garlic extract” (AGE) has been reported to have an array of pharmacological effects including: immunomodulation (1,1), tumor cell growth inhibition (12), anti-allergic effects (13), and chemo-preventative effects (7). A number of studies have suggested that AGE and its constituents could be a promising candidate as an immune modifier, which maintains the homeostasis of immune functions (14). Two major proteins, called 14 kDa and 47 kDa proteins, have been isolated and purified from AGE. Previous studies have shown that 14 kDa and 47 kDa proteins exert distinct effects on immune responses. Ghazanfari *et al* have reported that 47 kDa protein attenuated the delayed type hypersensitivity (DTH) response compared to either garlic extract and/or the 14 kDa protein extracted from AGE (9). Daneshmandi *et al* have also indicated that 47 kDa protein is able to suppress nitric oxide (NO) production by macrophages *in vitro*. Furthermore, 47 kDa protein was unable to induce augmented macrophage activity against WEHI-164 fibrosarcoma cells (15).

Dendritic cells (DCs) are known to be professional antigen-presenting cells (APCs) initiating immune responses. There is compelling evidence that DCs not only induce T cell activation, but also instigate peripheral T cell tolerance (16). These cells are present in almost all tissues in an immature state and in response to signals associated with infection (e.g. lipopolysaccharide (LPS)) or inflammation (e.g. TNF-α, interleukin-1β). Immature DCs can mature into potent antigen-presenting cells, which are characterized by the expression of high levels of MHC II, co-stimulatory molecules, pro-inflammatory cytokines, and induction of effective T cell responses (17).

According to the immunomodulatory properties of the 47 kDa protein isolated from AGE, we suppose that this protein can be a good candidate for generation tolerogenic DC *in vitro*. For this purpose, we investigated the effect of the 47 kDa protein isolated from AGE on mouse DCs *in vitro*. Based on the unique role of DCs in the induction of primary T cell responses, we analyzed the modulation of immature DCs phenotype by this protein. To do so, the surface expression of co-stimulatory molecules on DCs was examined. 

## Materials and Methods

***Animals***

Fifteen 8 to 10 week female inbred BALB/c mice were purchased from Pasteur Institute of Iran (Tehran, Iran). They were kept under optimal conditions of hygiene, temperature, humidity, and light (cycles of 12 hr dark/light). All experimental procedures on animals were approved by the Ethical Committee of the Tarbiat Modarres University, Tehran, Iran. 

***Purification of 47 kDa protein from AGE***

Fresh garlic bulbs were obtained from Hamadan, Iran. Dry garlic bulbs were peeled and kept for six months at -20 ºC. Aqueous AGE was prepared by the method used by Ghazanfari *et al* (10). Briefly, garlic samples were homogenized with two parts of distilled water in a varying blender. The homogenized blend was then filtered under vacuum through Wattman filter paper (number 1) and the filtrate was centrifuged at 3400×g for 30 min. The clear supernatant was collected. Twenty seven g of NH_4_SO_4 _(27 g) was added to 1 L of the supernatant and centrifuged at 3400×g for 30 min. The residue was subsequently resuspended in saline and dialyzed against buffer saline. Protein extract isolated from AGE was run through Amicon ultra filtration. The membrane used for this process was 20 pm (Millipore, USA). The remaining extracts after ultra filtration was collected as residues (R) 20. R20 was further purified by gel filtration chromatography with Sephadex G50. The purified protein was measured by Bradford assay and evaluated by SDS-PAGE.

***Evaluating the purity of 47 kDa protein by SDS-PAGE plectrophoresis***

A 12% (w/v) polyacrylamide gel was used to judge the purity of molecules and to estimate the molecular mass with standard protein. After electrophoresis, the gel was fixed with methanol and acetic acid formaldehyde for 60 min and stained with coomassie blue.

***Purification of splenic DCs***

A DC-enriched population was prepared from mice spleen according to the method reported by Vremec *et al* (18), with minor modifications. Briefly, after cervical dislocation, spleens were taken of *five BALB*/*c* mice under aseptic conditions in every experiment. Tissues were cut into small pieces with scissors, suspended in 5-10 ml RPMI-1640 (Gibco, UK) containing collagenase D (1 mg/ml; Roche, Germany) and DNase (0.02 mg/ml; Roche, Germany), and then digested for 30 min at 37 ºC in a 5% CO_2_ incubator. To disrupt cell aggregations or DC-T cell complexes, EDTA (5 mM, pH 7.2) was added at the end of incubation period and the cell suspension was pipetted several times. Undigested stromal fragments were afterwards removed by passing the suspension through a stainless steel sieve. The cell suspension was washed twice with phosphate-buffered saline (PBS) containing 5 mM EDTA at 4 ºC, 300×g for 10 min. The pellet was immediately resuspended in 2–3 ml RPMI and added slowly on 2 ml Nycodenz 13% (w/v), d= 1.068 (Axis-Shield, Norway) and centrifuged at 4 ºC, 600×g for 15 min. Low-density cells were recovered from the interface, washed twice with RPMI, and cultured in complete RPMI medium containing 5% fetal calf serum, non-essential amino acids, L-glutamine, penicillin, and streptomycin (all from Gibco, UK) for 120 min. Non-adherent cells were then removed by gently washing of the plates with warm RPMI and adherent cells were cultured for another 16-20 hr in complete RPMI medium. The total viable DC was determined by using the trypan blue exclusion assay.

***Treatment of DCs with 47 kDa protein purified from AGE***

To evaluate the effect of the 47 kDa protein isolated from AGE on the maturation of DCs, different concentrations of this protein were added to the overnight culture of DCs obtained from BALB/c mice. For this purpose, cells were divided into three groups. The first group was treated with the 47 kDa protein isolated from AGE, while the second and third groups were used as positive (Treated with TNF-α) and negative (not treated) controls, respectively. After removing the non-adherent cells, in order to optimize the concentration of 47 kDa protein needed for DC maturation, 2 × 10^5^ DCs were treated in 200 µl RPMI containing 0.5% normal mouse serum with different concentrations from 47 kDa protein including: 5, 10, and 20 μg/ml, during the overnight culture 2 × 10^5^ DCs were treated with RPMI 1640 containing 0.5% normal mouse serum in the absence of 47 kDa protein as the negative control group. The positive control group contained TNF-α (20ng/ml) treated 1 × 10^5^ DCs cultured in RPMI containing 0.5% normal mouse serum in the absence of 47 kDa protein.

After incubation time of 14-16 hr, the non-adherent cells (enriched DCs) were collected and analyzed using flowcytometry (Partec, Germany). In order to evaluate the inherent cytotoxicity effect of 47 kDa protein, DC viability after treatment with this protein was assessed by trypan blue exclusion assay. 

***Flowcytometric analysis***

In order to evaluate the purity of isolated DCs, and the effect of 47 kDa protein isolated from AGE on expression of DCs surface markers, DCs were stained for their phenotypic markers by a standard direct procedure using monoclonal antibodies. In brief, DCs were treated on ice with 5% normal hamster and rats serum for 15 min and then incubated with PE-conjugated anti-mouse CD11c (Pharmingen, Australia) diluted to 1 µg/10^6^ cell in PBS containing 2% FCS (PBS-FCS) for 30 min at 4 ºC. After being washed twice with PBS-FCS, DCs were incubated with one of the FITC-conjugated anti mouse; MHC-II, CD86, or CD40 (Pharmingen, Australia) for another 30 min. Cells were washed twice with PBS-FCS and re-suspended in 0.5 ml cold PBS–FCS and retained on ice until analysis with a flowcytometer (Partec, Germany). Appropriate isotype controls were used for all staining.

***Statistical analysis***

All data were presented as mean±SD and Mann-Whitney test was used in our experiments. *P* value ≤ 0.05 was regarded as statistically significant.

## Results

***Evaluation of the 47 kDa protein isolated from AGE***

**Figure 1:**
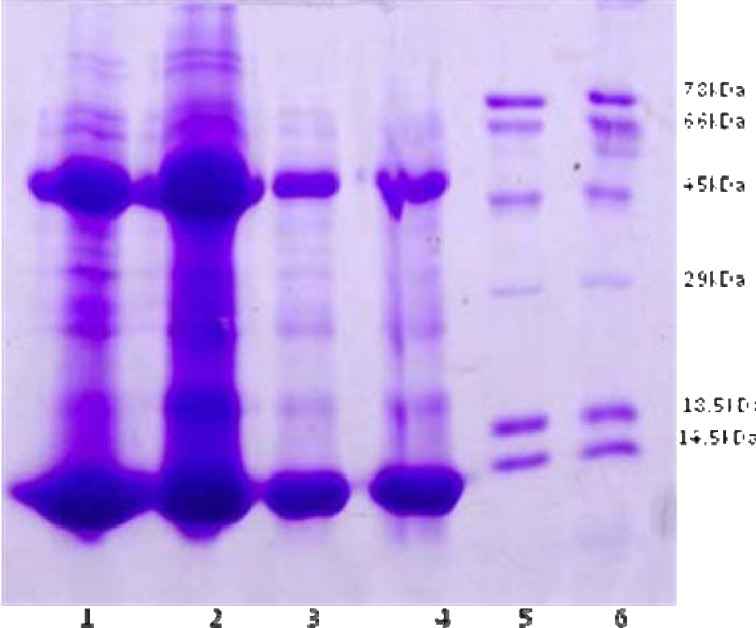
Evaluating the 47 kDa protein band in precipitated proteins from age garlic extract (AGE) by SDS-PAGE electrophoresis.

**Figure 2:**
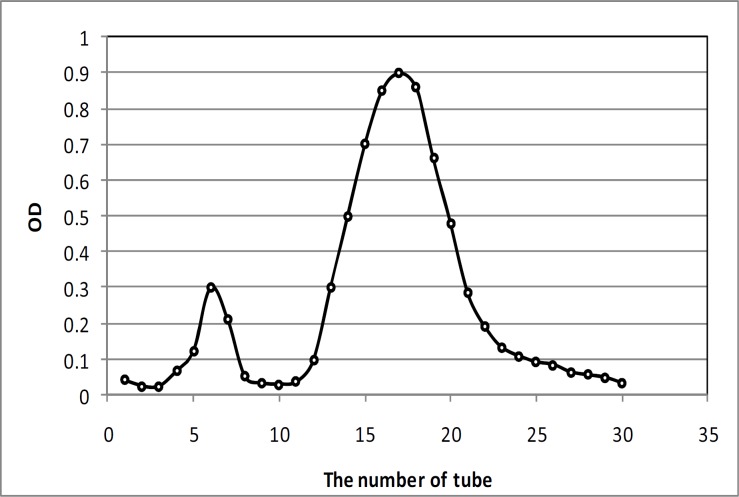
Purification and evaluation of 47 kDa proteins isolated from aged garlic extract (AGE).

Different proteins were purified from the AGE by ammonium sulfate precipitation (Figure 1) and in next step, protein solution as R20a Amicon ultra filtration was run on Sephadex G50 gel chromatography for further purification of the 47 kDa protein (Figure 2a). In order to evaluate the purity of 47 kDa protein, this protein was run on the SDS/PAGE electrophoresis and the results indicated the presence of 95% purified band of 47 kDa protein (Figure 2b). The purified 47 kDa protein concentration was 0.2 mg/ml as assessed by Bradford assay was 0.12 mg/ml. 

***Evaluation of the enriched DCs from mice***

In this study, 10-15×10^7^ mononuclear cells were obtained from each BALB/c mouse spleen and their viability was higher than 95%.

We used Nycodenz gradient medium with a density of 1.068 and short-term culture for enrichment of murine splenic DCs. The yield of low density cells in the first step of purification was 4–6% of starting cell population. After 2 hr of culture period and removing the non-adherent cells, only about 0.4% of all spleen cells remained attached to the plate. Overnight culture, resulting in maturation of DCs, caused these cells to be floated. Floating cells were stained for DC specific marker by a standard direct procedure using PE-conjugated anti-mouse CD11c. Results showed that yield of purified DCs from each mouse spleen was 3–6×10^5^ cells with more than 90% purity (94.5±3.2%). 

**Figure 3:**
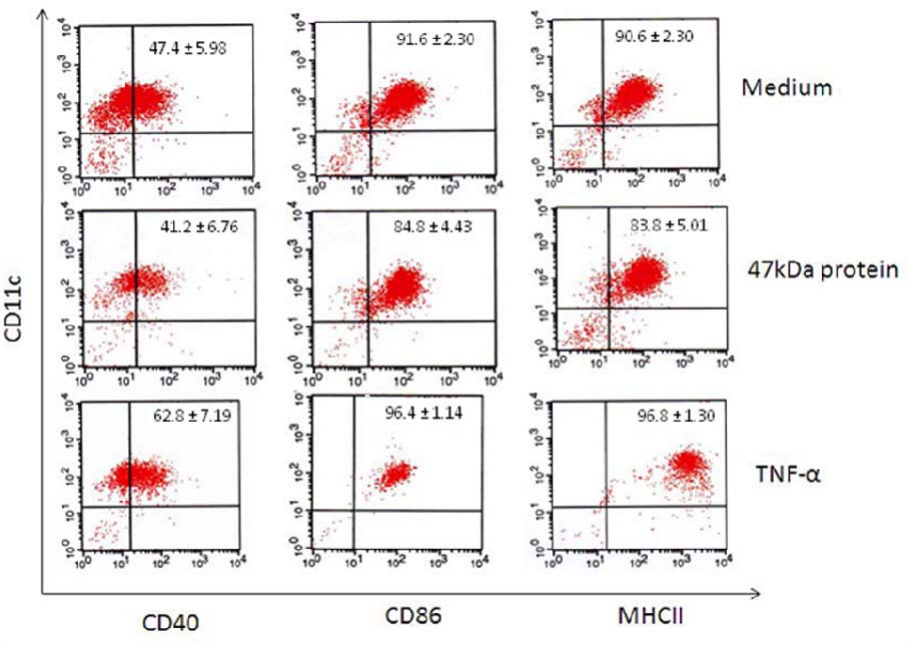
Cell-surface expression of MHC-II and costimulatory molecules on Dendritic cells (DCs) upon addition of 47 kDa protein or TNF-α. Immature DCs were not treated or treated with 47 kDa protein purified from aged garlic extract (AGE) or with TNF-α (20ng/ml) for 14-16 h, stained with PE-conjugated mAb to CD11c and FITC-conjugated mAb to CD40, CD86, or MHC- II and then examined by flow cytometry. The dot-plots show representative data out of five independent experiments. Data presented in the upper right show mean percentage of double-positive cells±S.D.

***Effect of 47 kDa on expression of DC maturation markers***

To evaluate the maturation of DCs, 5 μg/ml of 47 kDa purified protein of AGE was added to 2×10^5^ DCs in 200 µl RPMI containing normal mouse serum and cultured overnight. The results of flowcytometric analysis using double staining of CD11c and CD40, CD86 or MHC-II showed that 47 kDa protein decrease surface expression of, CD40, CD86 and MHC-II on treated DCs in comparison with non-treated DCs (median of 41% versus 47%, 84% versus 91% and 83% versus 90%, respectively); but we observed no statistical difference between the two groups (Figure 3). Also results of mean fluorescence intensity (MFI) weren’t significant for treated and non-treated DCs. However, we showed that, in comparison with 14 kDa protein isolated from AGE, 47 kDa protein lowered significantly (*P*<0.05) the expression of CD40, CD86 and MHC-II on DCs (data not shown). The viability percentage of DC was similar in treated and untreated cells (more than 90% viable as assessed by trypan blue exclusion assay.)

## Discussion

In this study, we evaluated the effect of the 47 kDa protein isolated from AGE on mouse DCs. Our results showed that 47 kDa protein-treated DCs decreases the expression of DC maturation markers including: CD40, CD86 and MHC-II in comparison with non- treated DCs, however, the difference was not statistically significant. However, 47 kDa protein in comparison with 14 kDa protein isolated from AGE significantly decreased (*P*<0.05) the expression of CD40, CD86 and MHC-II on DCs (data not shown).

*In vitro* treatment of DCs with different immunomodulatory components can enhance and stabilize DCs tolerogenic properties (17, 19). Numerous reports suggest that such immature, maturation-resistant or “alternatively activated” DCs can regulate autoreactive or alloreactive T-cell responses and be used as an immunotherapeutic tool for treating diseases characterized by a break-down in immune tolerance (20). Recently, researchers have made great effort to find a potent mediator to generate stable tolerogenic DC *in vitro* and *in vivo*.

Previous studies have shown that 47 kDa protein isolated from AGE possesses a number of immunomedulatory effects. The 47 kDa protein isolated from AGE attenuated DTH response and suppressed nitric oxide (NO) production by macrophages (9, 15). Also this protein was not able to induce augmented macrophage activity against WEHI-164 fibrosarcoma cells (15).

 Tolerogenic DCs have a typical tolerogenic phenotype with low expression of co-stimulatory molecules and an anti-inflammatory cytokine profile (21). Numerous reports suggest that tolerogenic DCs can regulate both autoreactive and alloreactive T-cell responses and also induce antigen-specific tolerance in experimental animal models (22-24). Tolerogenic DCs show great promise as a cellular tolerogenic tool for the treatment of autoimmune disorders (16). In recent years, scientists have tried to find a simple, cheap and effective method for production of tolerogenic DC. To this end, they evaluated the effect of different mediators such as dexamethasone and 1,25-(OH)_2_D_3_ on DCs function and phenotype (25). However, using such immature DCs for the treatment of autoimmune disorders may be unsafe, as immature DCs are not stable. They can lose their tolerogenic ability and become immunogenic in response to different factors including: pro-inflammatory cytokines (e.g. TNF-α, IL-1) (26). According to the results of the present study, we found that the 47 kDa protein purified from AGE can be considered as a candidate to generate tolerogenic DC. Therefore, the 47 kDa protein of plant origin may be a safe, stable and simple method compared with pharmaceutical compounds (such as dexamethasone and 1,25-(OH)_2_D_3_) to tolerogenic DC generation *in vitro*. To our knowledge, data regarding the potential effects of different protein components of garlic on DCs either functional or phenotypic maturation is still elusive. This is the first study to explore the potential effect of the garlic 47 kDa protein on DC maturation, however determining precise mechanism involved in this effect calls for further investigations.

In future studies, we plan to evaluate the effect of the 47 kDa protein isolated from AGE on DCs function and clarify the mechanism through which this protein component affects DCs. Future studies should also focus on application of 47 kDa treated DC in animal models of autoimmune disease. 

## Conclusion

According to the present study, the 47 kDa protein purified from AGE can be regarded as a potential candidate for generating tolerogenic DC *in vitro*. Such DC could be further utilized as an effective way for inducing tolerance in autoimmune diseases.
